# 3,3′-Dibromo-4,4′-[(1*R*,2*R*)-cyclohexane-1,2-diyldiimino]dipent-3-en-2-one

**DOI:** 10.1107/S1600536808043213

**Published:** 2008-12-24

**Authors:** Yun-Qian Zhang, Qi-Long Zhang, Bi-Xue Zhu

**Affiliations:** a Key Laboratory of Macrocyclic and Supramolecular Chemistry, of Guizhou Province, Guizhou University, Guiyang, 550025, People’s Republic of China.

## Abstract

The asymmetric unit of the title compound, C_16_H_24_Br_2_N_2_O_2_, contains two independent mol­ecules, each which has two intra­molecular N—H⋯O hydrogen bonds linking the amine N atoms to the enolic O atoms of the same acacH-imine unit. In the crystal, the mol­ecules are lined up by inter­molecular weak C—H⋯O hydrogen bonds, forming two vertical each other two-dimensional chains along the *a* axis and *b* axis of the unit cell, respectively.

## Related literature

For general background, see: Bottcher *et al.* (1997 ▶); Bu *et al.* (1997 ▶); Chimpalee *et al.* (2000 ▶); Dominiak *et al.* (2003 ▶); Gilli *et al.* (1989 ▶); McCann *et al.* (2001 ▶); Na *et al.* (2002 ▶); Ozkar *et al.* (2004 ▶); Tacke *et al.* (2003 ▶); Zhang *et al.* (2003 ▶).
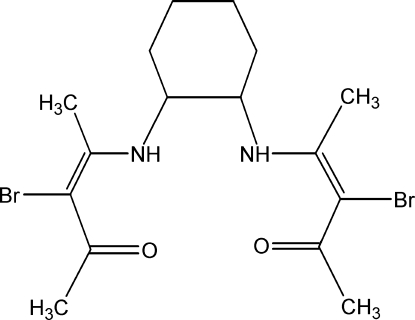


## Experimental

### 

#### Crystal data

C_16_H_24_Br_2_N_2_O_2_
*M* *_r_* = 436.19
Monoclinic, 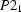

*a* = 9.249 (5) Å
*b* = 9.350 (6) Å
*c* = 21.82 (2) Å
β = 99.122 (13)°
*V* = 1863 (3) Å^3^
*Z* = 4
Mo *K*α radiation
μ = 4.36 mm^−1^
*T* = 298 (2) K
0.21 × 0.18 × 0.16 mm

#### Data collection

Bruker APEXII CCD area-detector diffractometer
Absorption correction: multi-scan (*SADABS*; Bruker, 2005 ▶) *T*_min_ = 0.461, *T*_max_ = 0.542 (expected range = 0.424–0.498)
12101 measured reflections
3433 independent reflections
1894 reflections with *I* > 2σ(*I*)
*R*_int_ = 0.055

#### Refinement

*R*[*F* ^2^ > 2σ(*F* ^2^)] = 0.042
*wR*(*F* ^2^) = 0.096
*S* = 0.97
3433 reflections
405 parameters
1 restraint
H-atom parameters constrained
Δρ_max_ = 0.38 e Å^−3^
Δρ_min_ = −0.42 e Å^−3^

### 

Data collection: *APEX2* (Bruker, 2005 ▶); cell refinement: *SAINT* (Bruker, 2005 ▶); data reduction: *SAINT*; program(s) used to solve structure: *SHELXS97* (Sheldrick, 2008 ▶); program(s) used to refine structure: *SHELXL97* (Sheldrick, 2008 ▶); molecular graphics: *ORTEP-3 for Windows* (Farrugia, 1997 ▶); software used to prepare material for publication: *WinGX* (Farrugia, 1999 ▶).

## Supplementary Material

Crystal structure: contains datablocks global, I. DOI: 10.1107/S1600536808043213/at2694sup1.cif

Structure factors: contains datablocks I. DOI: 10.1107/S1600536808043213/at2694Isup2.hkl

Additional supplementary materials:  crystallographic information; 3D view; checkCIF report

## Figures and Tables

**Table 1:** Hydrogen-bond geometry (Å, °)

*D*—H⋯*A*	*D*—H	H⋯*A*	*D*⋯*A*	*D*—H⋯*A*
N1—H1⋯O1	0.86	1.96	2.588 (8)	129
N2—H2⋯O2	0.86	1.93	2.584 (9)	131
N3—H3⋯O3	0.86	1.98	2.602 (9)	129
N4—H4⋯O4	0.86	1.97	2.596 (9)	129
C5—H5*C*⋯O2^i^	0.96	2.66	3.463 (12)	142
C12—H12*B*⋯O1^ii^	0.96	2.56	3.416 (12)	149
C23—H23*A*⋯O3^iii^	0.97	2.66	3.581 (12)	159
C28—H28*C*⋯O4^iv^	0.96	2.65	3.419 (13)	138

Symmetry codes: (i) 
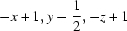
; (ii) 
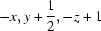
; (iii) 
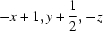
; (iv) 
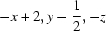
.

**Fig. 1.**
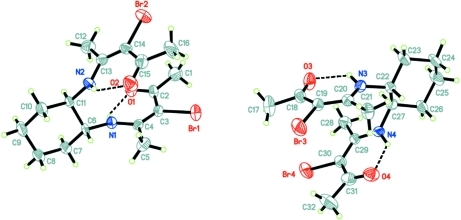
The molecular structure of (II) showing the atom-labelling scheme. Displacement ellipsoids are drawn at the 50% probability level. Dashed lines indicate hydrogen bonds.

**Fig. 2.**
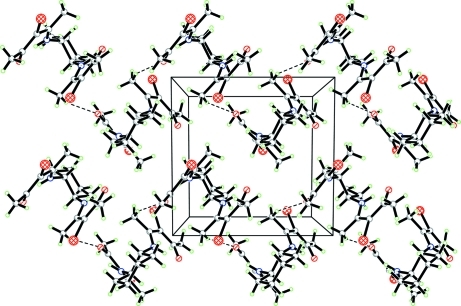
Packing diagram of (II), viewed in the ab plane, with the C—H···O interactions shown as dashed lines.

## supplementary crystallographic information

### Comment

Schiff base obtained from condensation of acetylacetone and different diamines have been used as ligand for the complex formation with a variety of transition metals (Bottcher *et al.*, 1997; McCann *et al.*, 2001; Na *et al.*, 2002; Ozkar *et al.*, 2004; Tacke *et al.*, 2003). and have found immense analytical applications (Chimpalee *et al.*, 2000; Zhang *et al.*, 2003). In this work, we report a crystal structure of N, *N*'-bis(bromo-acetylacetone)-1*R*,2*R*-diaminocyclohexane ligands.

The crystal structure of the title compound is shown in Fig. 1, each dissymmetrical unit cell contains two vertical each other independent molecules. Each molecule has two intramolecular N^+^—H···O^-^ hydrogen bonds, which links each nitrogen atoms to the corresponding nearby terminal oxygen atoms of the same acacH-imine unit (N1—H1···O1, N2—H2···O2, N3—H3···O3 and N4—H4···O4, Table 1) such that a coplanar six-membered ring is generated. As shown in Fig. 2, the molecules of the title compound are lined up by the intermolecular interaction (C—H···O, Table 1.) forming two vertical each other two-dimensional chains along the *a* axis and *b* axis of the unit cell, respectively. The structure also shows a non-coplanar array for the (*R*, *R*)-cyclohexanediamine moiety and both of the C=N imine groups have the *Z* arrangements with respect to the chiral C—C sigma bond (C6—C11 or C22—C27) in the cyclohexanediamine, and the Schiff base molecule are non-coplanar due to chirality of the cyclohexanediamine moiety.

### Experimental

1*R*,2*R*-Diaminocyclohexane (0.115 g, 1.00 mmol) was added slowly, whilst stirring, to a methanol (15 ml) solution with acetylacetone (0.2 g, 2.00 mmol), and the mixture was heated at reflux for 2 h. After cooling, and the solvent was removed under reduced pressure. The crude product was purified by column chromatography over silica gel using 20% EtOAc-hexane to afford pure yellow crystals of *N*,*N*'-bis-acetylacetone-1*R*,2*R*-dDiaminocyclohexane and dried in vacuum. Solid *N*-bromosuccimide (0.088 g, 0.5 mmol) was added slowly, whilst stirring, to a solution of the compound 1 (0.14 g, 0.5 mmol) in ethanol (20 ml). Stirring the solution for 2 h, and then the solvent was removed under reduced pressure. The crude product was purified by column chromatography over silica gel using 35% EtOAc-CH_2_Cl_2_ to afford pure pale yellow crystals of 2 and dried in vacuum, 0.1 g (yield 46%). Single crystals suitable for X-ray diffraction were obtained from an ethanol-CH_2_Cl_2_ mixture by slow evaporation at room temperature.

### Refinement

All H atoms were placed in calculated positions and refined as riding, with C—H = 0.96-0.98 Å, N—H = 0.86 Å, and *U*_iso_(H) = 1.2–1.5*U*_eq_(C,*N*).

### Crystal data

**Table:**

C_16_H_24_Br_2_N_2_O_2_	*F*(000) = 880
*M**_r_* = 436.19	*D*_x_ = 1.555 Mg m^−^^3^
Monoclinic, *P*2_1_	Mo *K*α radiation, λ = 0.71073 Å
Hall symbol: P 2yb	Cell parameters from 5587 reflections
*a* = 9.249 (5) Å	θ = 1.0–25.0°
*b* = 9.350 (6) Å	µ = 4.36 mm^−^^1^
*c* = 21.82 (2) Å	*T* = 298 K
β = 99.122 (13)°	Prism, colourless
*V* = 1863 (3) Å^3^	0.21 × 0.18 × 0.16 mm
*Z* = 4	

### Data collection

**Table:**

Bruker APEXII CCD area-detector diffractometer	3433 independent reflections
Radiation source: fine-focus sealed tube	1894 reflections with *I* > 2σ(*I*)
graphite	*R*_int_ = 0.055
φ and ω scan	θ_max_ = 25.0°, θ_min_ = 1.0°
Absorption correction: multi-scan (*SADABS*; Bruker, 2005)	*h* = −10→10
*T*_min_ = 0.461, *T*_max_ = 0.542	*k* = −9→10
12101 measured reflections	*l* = −25→25

### Refinement

**Table:**

Refinement on *F*^2^	Primary atom site location: structure-invariant direct methods
Least-squares matrix: full	Secondary atom site location: difference Fourier map
*R*[*F*^2^ > 2σ(*F*^2^)] = 0.042	Hydrogen site location: inferred from neighbouring sites
*wR*(*F*^2^) = 0.096	H-atom parameters constrained
*S* = 0.97	*w* = 1/[σ^2^(*F*_o_^2^) + (0.0413*P*)^2^] where *P* = (*F*_o_^2^ + 2*F*_c_^2^)/3
3433 reflections	(Δ/σ)_max_ < 0.001
405 parameters	Δρ_max_ = 0.38 e Å^−^^3^
1 restraint	Δρ_min_ = −0.42 e Å^−^^3^

### Special details

**Table:**

Geometry. All e.s.d.'s (except the e.s.d. in the dihedral angle between two l.s. planes) are estimated using the full covariance matrix. The cell e.s.d.'s are taken into account individually in the estimation of e.s.d.'s in distances, angles and torsion angles; correlations between e.s.d.'s in cell parameters are only used when they are defined by crystal symmetry. An approximate (isotropic) treatment of cell e.s.d.'s is used for estimating e.s.d.'s involving l.s. planes.
Refinement. Refinement of *F*^2^ against ALL reflections. The weighted *R*-factor *wR* and goodness of fit *S* are based on *F*^2^, conventional *R*-factors *R* are based on *F*, with *F* set to zero for negative *F*^2^. The threshold expression of *F*^2^ > σ(*F*^2^) is used only for calculating *R*-factors(gt) *etc*. and is not relevant to the choice of reflections for refinement. *R*-factors based on *F*^2^ are statistically about twice as large as those based on *F*, and *R*- factors based on ALL data will be even larger.

### Fractional atomic coordinates and isotropic or equivalent isotropic displacement parameters (Å^2^)

**Table:**

	*x*	*y*	*z*	*U*_iso_*/*U*_eq_	
C1	0.1762 (11)	0.3858 (14)	0.3222 (5)	0.102 (4)	
H1A	0.2497	0.3346	0.3045	0.153*	
H1B	0.1468	0.4689	0.2975	0.153*	
H1C	0.0929	0.3250	0.3230	0.153*	
C16	0.1713 (12)	0.9661 (18)	0.3226 (5)	0.131 (5)	
H16A	0.0735	0.9879	0.3029	0.197*	
H16B	0.2099	0.8896	0.3007	0.197*	
H16C	0.2319	1.0493	0.3221	0.197*	
C17	0.5609 (13)	0.7298 (16)	0.1865 (4)	0.115 (4)	
H17A	0.4725	0.7689	0.1975	0.172*	
H17B	0.6440	0.7704	0.2127	0.172*	
H17C	0.5612	0.6279	0.1919	0.172*	
C32	1.1245 (13)	0.7174 (16)	0.1748 (4)	0.122 (5)	
H32A	1.2105	0.7738	0.1732	0.183*	
H32B	1.1522	0.6277	0.1947	0.183*	
H32C	1.0604	0.7677	0.1979	0.183*	
C2	0.2376 (11)	0.4310 (12)	0.3872 (4)	0.069 (3)	
C3	0.3634 (9)	0.5293 (10)	0.4000 (4)	0.055 (2)	
C4	0.4082 (8)	0.5911 (10)	0.4549 (4)	0.046 (2)	
C5	0.5318 (9)	0.6972 (11)	0.4656 (4)	0.070 (3)	
H5A	0.4926	0.7922	0.4662	0.106*	
H5B	0.5895	0.6896	0.4329	0.106*	
H5C	0.5922	0.6776	0.5047	0.106*	
C6	0.3513 (9)	0.6388 (10)	0.5616 (3)	0.052 (2)	
H6	0.4254	0.7139	0.5619	0.062*	
C7	0.3927 (9)	0.5475 (11)	0.6202 (4)	0.070 (3)	
H7A	0.3207	0.4722	0.6209	0.083*	
H7B	0.4872	0.5029	0.6196	0.083*	
C8	0.3994 (10)	0.6376 (11)	0.6772 (4)	0.071 (3)	
H8A	0.4779	0.7067	0.6782	0.085*	
H8B	0.4220	0.5771	0.7136	0.085*	
C9	0.2550 (10)	0.7176 (12)	0.6800 (4)	0.077 (3)	
H9A	0.2673	0.7807	0.7157	0.092*	
H9B	0.1785	0.6493	0.6847	0.092*	
C10	0.2096 (10)	0.8057 (11)	0.6204 (4)	0.072 (3)	
H10A	0.1151	0.8499	0.6215	0.086*	
H10B	0.2805	0.8813	0.6183	0.086*	
C11	0.2002 (8)	0.7125 (9)	0.5630 (3)	0.043 (2)	
H11	0.1257	0.6386	0.5646	0.052*	
C12	−0.0578 (9)	0.6500 (11)	0.4709 (4)	0.078 (3)	
H12A	−0.1318	0.6411	0.4349	0.117*	
H12B	−0.1019	0.6817	0.5055	0.117*	
H12C	−0.0119	0.5589	0.4804	0.117*	
C13	0.0560 (8)	0.7578 (10)	0.4581 (4)	0.049 (2)	
C14	0.0573 (9)	0.8253 (10)	0.4022 (4)	0.057 (2)	
C15	0.1691 (11)	0.9218 (11)	0.3882 (5)	0.067 (3)	
C18	0.5690 (10)	0.7649 (12)	0.1194 (4)	0.065 (3)	
C19	0.6550 (9)	0.8842 (10)	0.1036 (4)	0.051 (2)	
C20	0.6869 (9)	0.9063 (9)	0.0454 (4)	0.048 (2)	
C21	0.7836 (10)	1.0251 (10)	0.0312 (4)	0.069 (3)	
H21A	0.8836	1.0023	0.0474	0.103*	
H21B	0.7563	1.1118	0.0501	0.103*	
H21C	0.7731	1.0379	−0.0129	0.103*	
C22	0.6848 (8)	0.8009 (8)	−0.0591 (4)	0.042 (2)	
H22	0.7621	0.8716	−0.0611	0.051*	
C23	0.5697 (10)	0.8218 (10)	−0.1152 (4)	0.066 (3)	
H23A	0.5299	0.9176	−0.1146	0.079*	
H23B	0.4905	0.7545	−0.1138	0.079*	
C24	0.6317 (11)	0.8001 (12)	−0.1745 (4)	0.077 (3)	
H24A	0.5537	0.8106	−0.2096	0.093*	
H24B	0.7042	0.8735	−0.1777	0.093*	
C25	0.7009 (12)	0.6564 (15)	−0.1775 (4)	0.100 (4)	
H25A	0.7474	0.6501	−0.2143	0.120*	
H25B	0.6268	0.5824	−0.1799	0.120*	
C26	0.8189 (10)	0.6342 (11)	−0.1174 (4)	0.069 (3)	
H26A	0.8597	0.5387	−0.1179	0.083*	
H26B	0.8980	0.7021	−0.1177	0.083*	
C27	0.7536 (9)	0.6538 (9)	−0.0592 (4)	0.053 (2)	
H27	0.6771	0.5817	−0.0583	0.064*	
C28	0.7431 (10)	0.4321 (10)	0.0342 (5)	0.069 (3)	
H28A	0.6476	0.4693	0.0370	0.104*	
H28B	0.7692	0.3612	0.0659	0.104*	
H28C	0.7423	0.3894	−0.0058	0.104*	
C29	0.8524 (8)	0.5509 (9)	0.0430 (4)	0.048 (2)	
C30	0.9422 (9)	0.5755 (10)	0.0989 (4)	0.057 (2)	
C31	1.0469 (10)	0.6904 (13)	0.1100 (5)	0.071 (3)	
N1	0.3468 (6)	0.5565 (7)	0.5046 (3)	0.0480 (18)	
H1	0.2996	0.4770	0.5026	0.058*	
N2	0.1623 (6)	0.7941 (8)	0.5063 (3)	0.0534 (18)	
H2	0.2105	0.8715	0.5028	0.064*	
N3	0.6301 (6)	0.8226 (7)	−0.0014 (3)	0.0495 (18)	
H3	0.5522	0.7761	0.0032	0.059*	
N4	0.8647 (6)	0.6349 (7)	−0.0047 (3)	0.0509 (19)	
H4	0.9450	0.6820	−0.0034	0.061*	
O1	0.1794 (7)	0.3886 (7)	0.4302 (3)	0.0709 (18)	
O2	0.2644 (7)	0.9658 (7)	0.4308 (3)	0.0779 (19)	
O3	0.5019 (7)	0.6859 (8)	0.0789 (3)	0.085 (2)	
O4	1.0671 (6)	0.7704 (8)	0.0690 (3)	0.081 (2)	
Br1	0.44737 (12)	0.58781 (14)	0.32986 (5)	0.0905 (4)	
Br2	−0.08805 (11)	0.76970 (15)	0.33409 (5)	0.0971 (4)	
Br3	0.74344 (13)	1.00453 (13)	0.17037 (5)	0.0946 (4)	
Br4	0.91939 (13)	0.45712 (13)	0.16842 (5)	0.0993 (5)	

### Atomic displacement parameters (Å^2^)

**Table:**

	*U*^11^	*U*^22^	*U*^33^	*U*^12^	*U*^13^	*U*^23^
C1	0.082 (8)	0.137 (11)	0.085 (8)	0.005 (7)	0.008 (6)	−0.040 (8)
C16	0.119 (10)	0.208 (16)	0.064 (8)	−0.021 (11)	0.011 (7)	0.071 (9)
C17	0.160 (11)	0.127 (11)	0.067 (7)	−0.012 (9)	0.050 (7)	0.005 (8)
C32	0.131 (9)	0.147 (13)	0.074 (8)	0.010 (9)	−0.029 (7)	−0.006 (9)
C2	0.074 (7)	0.091 (8)	0.043 (6)	0.012 (6)	0.017 (5)	−0.001 (6)
C3	0.054 (5)	0.066 (7)	0.046 (6)	0.009 (5)	0.015 (4)	0.003 (5)
C4	0.042 (5)	0.048 (5)	0.050 (5)	0.007 (5)	0.013 (4)	0.003 (5)
C5	0.059 (6)	0.069 (7)	0.086 (7)	−0.004 (5)	0.022 (5)	−0.012 (6)
C6	0.064 (6)	0.069 (7)	0.024 (4)	−0.001 (5)	0.012 (4)	0.001 (4)
C7	0.071 (6)	0.091 (9)	0.046 (6)	0.022 (6)	0.007 (4)	0.012 (6)
C8	0.081 (7)	0.087 (8)	0.042 (6)	0.017 (6)	0.000 (5)	0.011 (5)
C9	0.103 (7)	0.091 (8)	0.037 (5)	0.016 (7)	0.011 (5)	−0.001 (5)
C10	0.083 (6)	0.082 (8)	0.050 (6)	0.007 (6)	0.010 (5)	−0.007 (6)
C11	0.051 (5)	0.051 (5)	0.028 (4)	−0.002 (4)	0.006 (4)	0.001 (4)
C12	0.061 (6)	0.086 (9)	0.085 (8)	−0.017 (6)	0.007 (5)	−0.011 (6)
C13	0.050 (5)	0.044 (6)	0.052 (6)	0.002 (5)	0.009 (4)	−0.005 (5)
C14	0.049 (5)	0.066 (7)	0.052 (6)	0.003 (5)	−0.002 (4)	−0.013 (5)
C15	0.076 (7)	0.061 (7)	0.065 (7)	0.006 (6)	0.017 (6)	0.004 (6)
C18	0.082 (6)	0.067 (7)	0.049 (6)	0.023 (7)	0.018 (5)	0.002 (6)
C19	0.053 (5)	0.053 (6)	0.047 (6)	−0.010 (5)	0.008 (4)	−0.011 (5)
C20	0.052 (5)	0.039 (6)	0.052 (6)	0.007 (4)	0.008 (5)	−0.002 (5)
C21	0.093 (7)	0.060 (7)	0.056 (6)	−0.015 (6)	0.018 (5)	−0.008 (5)
C22	0.051 (5)	0.036 (6)	0.039 (5)	−0.006 (4)	0.004 (4)	−0.003 (4)
C23	0.092 (7)	0.064 (7)	0.039 (5)	0.015 (5)	0.001 (5)	0.001 (5)
C24	0.104 (7)	0.089 (8)	0.034 (6)	0.034 (7)	−0.003 (5)	0.011 (5)
C25	0.120 (9)	0.144 (12)	0.034 (6)	0.042 (8)	0.001 (6)	−0.024 (6)
C26	0.087 (7)	0.079 (8)	0.041 (5)	0.016 (5)	0.008 (5)	−0.002 (5)
C27	0.049 (5)	0.060 (7)	0.050 (6)	−0.008 (4)	0.005 (4)	−0.006 (5)
C28	0.079 (7)	0.051 (7)	0.075 (7)	−0.008 (5)	0.006 (5)	0.018 (6)
C29	0.049 (5)	0.051 (7)	0.044 (5)	0.010 (5)	0.010 (4)	0.002 (5)
C30	0.064 (5)	0.052 (6)	0.053 (6)	0.006 (5)	0.005 (5)	0.003 (5)
C31	0.069 (7)	0.063 (8)	0.076 (8)	0.001 (6)	0.000 (6)	−0.003 (7)
N1	0.052 (4)	0.048 (5)	0.045 (4)	−0.013 (3)	0.012 (3)	−0.004 (4)
N2	0.058 (4)	0.058 (5)	0.041 (4)	−0.007 (4)	−0.002 (3)	0.003 (4)
N3	0.047 (4)	0.056 (5)	0.046 (4)	−0.012 (3)	0.009 (3)	−0.012 (4)
N4	0.039 (4)	0.058 (5)	0.054 (5)	−0.007 (3)	0.003 (3)	0.008 (4)
O1	0.084 (4)	0.077 (5)	0.053 (4)	−0.013 (4)	0.015 (4)	−0.009 (4)
O2	0.084 (5)	0.084 (5)	0.065 (4)	−0.019 (4)	0.009 (4)	0.013 (4)
O3	0.101 (5)	0.091 (6)	0.068 (5)	−0.035 (4)	0.033 (4)	−0.007 (4)
O4	0.070 (4)	0.075 (5)	0.093 (5)	−0.015 (4)	−0.002 (4)	0.011 (5)
Br1	0.1002 (8)	0.1210 (10)	0.0566 (7)	0.0003 (8)	0.0317 (6)	0.0100 (7)
Br2	0.0836 (7)	0.1347 (11)	0.0628 (7)	0.0004 (8)	−0.0196 (5)	−0.0041 (8)
Br3	0.1140 (9)	0.1142 (10)	0.0531 (6)	−0.0114 (8)	0.0055 (6)	−0.0306 (7)
Br4	0.1200 (10)	0.1090 (11)	0.0666 (8)	0.0057 (8)	0.0076 (6)	0.0326 (7)

### Geometric parameters (Å, °)

**Table:**

C1—C2	1.503 (13)	C12—H12C	0.9600
C1—H1A	0.9600	C13—N2	1.364 (9)
C1—H1B	0.9600	C13—C14	1.376 (11)
C1—H1C	0.9600	C14—C15	1.442 (13)
C16—C15	1.492 (13)	C14—Br2	1.911 (8)
C16—H16A	0.9600	C15—O2	1.246 (11)
C16—H16B	0.9600	C18—O3	1.240 (11)
C16—H16C	0.9600	C18—C19	1.444 (14)
C17—C18	1.513 (12)	C19—C20	1.365 (11)
C17—H17A	0.9600	C19—Br3	1.917 (8)
C17—H17B	0.9600	C20—N3	1.327 (10)
C17—H17C	0.9600	C20—C21	1.489 (12)
C32—C31	1.501 (12)	C21—H21A	0.9600
C32—H32A	0.9600	C21—H21B	0.9600
C32—H32B	0.9600	C21—H21C	0.9600
C32—H32C	0.9600	C22—N3	1.445 (10)
C2—O1	1.220 (10)	C22—C23	1.502 (10)
C2—C3	1.474 (13)	C22—C27	1.515 (11)
C3—C4	1.335 (11)	C22—H22	0.9800
C3—Br1	1.903 (8)	C23—C24	1.510 (12)
C4—N1	1.341 (9)	C23—H23A	0.9700
C4—C5	1.504 (12)	C23—H23B	0.9700
C5—H5A	0.9600	C24—C25	1.494 (14)
C5—H5B	0.9600	C24—H24A	0.9700
C5—H5C	0.9600	C24—H24B	0.9700
C6—N1	1.457 (9)	C25—C26	1.581 (11)
C6—C7	1.536 (11)	C25—H25A	0.9700
C6—C11	1.564 (10)	C25—H25B	0.9700
C6—H6	0.9800	C26—C27	1.502 (10)
C7—C8	1.495 (11)	C26—H26A	0.9700
C7—H7A	0.9700	C26—H26B	0.9700
C7—H7B	0.9700	C27—N4	1.453 (9)
C8—C9	1.541 (12)	C27—H27	0.9800
C8—H8A	0.9700	C28—C29	1.494 (12)
C8—H8B	0.9700	C28—H28A	0.9600
C9—C10	1.541 (11)	C28—H28B	0.9600
C9—H9A	0.9700	C28—H28C	0.9600
C9—H9B	0.9700	C29—N4	1.324 (9)
C10—C11	1.516 (11)	C29—C30	1.381 (11)
C10—H10A	0.9700	C30—C31	1.441 (14)
C10—H10B	0.9700	C30—Br4	1.916 (9)
C11—N2	1.448 (9)	C31—O4	1.204 (11)
C11—H11	0.9800	N1—H1	0.8600
C12—C13	1.515 (12)	N2—H2	0.8600
C12—H12A	0.9600	N3—H3	0.8600
C12—H12B	0.9600	N4—H4	0.8600
			
C2—C1—H1A	109.5	C13—C14—C15	125.7 (8)
C2—C1—H1B	109.5	C13—C14—Br2	117.4 (7)
H1A—C1—H1B	109.5	C15—C14—Br2	116.4 (7)
C2—C1—H1C	109.5	O2—C15—C14	119.6 (9)
H1A—C1—H1C	109.5	O2—C15—C16	120.6 (10)
H1B—C1—H1C	109.5	C14—C15—C16	119.8 (10)
C15—C16—H16A	109.5	O3—C18—C19	121.5 (8)
C15—C16—H16B	109.5	O3—C18—C17	117.7 (11)
H16A—C16—H16B	109.5	C19—C18—C17	120.8 (10)
C15—C16—H16C	109.5	C20—C19—C18	123.2 (8)
H16A—C16—H16C	109.5	C20—C19—Br3	119.3 (7)
H16B—C16—H16C	109.5	C18—C19—Br3	117.2 (7)
C18—C17—H17A	109.5	N3—C20—C19	120.6 (8)
C18—C17—H17B	109.5	N3—C20—C21	117.3 (8)
H17A—C17—H17B	109.5	C19—C20—C21	122.1 (8)
C18—C17—H17C	109.5	C20—C21—H21A	109.5
H17A—C17—H17C	109.5	C20—C21—H21B	109.5
H17B—C17—H17C	109.5	H21A—C21—H21B	109.5
C31—C32—H32A	109.5	C20—C21—H21C	109.5
C31—C32—H32B	109.5	H21A—C21—H21C	109.5
H32A—C32—H32B	109.5	H21B—C21—H21C	109.5
C31—C32—H32C	109.5	N3—C22—C23	113.0 (7)
H32A—C32—H32C	109.5	N3—C22—C27	109.5 (7)
H32B—C32—H32C	109.5	C23—C22—C27	111.1 (7)
O1—C2—C3	119.2 (8)	N3—C22—H22	107.7
O1—C2—C1	119.2 (10)	C23—C22—H22	107.7
C3—C2—C1	121.5 (9)	C27—C22—H22	107.7
C4—C3—C2	124.3 (8)	C22—C23—C24	111.4 (7)
C4—C3—Br1	119.3 (7)	C22—C23—H23A	109.4
C2—C3—Br1	116.0 (7)	C24—C23—H23A	109.4
C3—C4—N1	120.7 (8)	C22—C23—H23B	109.4
C3—C4—C5	123.1 (8)	C24—C23—H23B	109.3
N1—C4—C5	116.2 (7)	H23A—C23—H23B	108.0
C4—C5—H5A	109.5	C25—C24—C23	112.4 (8)
C4—C5—H5B	109.5	C25—C24—H24A	109.1
H5A—C5—H5B	109.5	C23—C24—H24A	109.1
C4—C5—H5C	109.5	C25—C24—H24B	109.1
H5A—C5—H5C	109.5	C23—C24—H24B	109.1
H5B—C5—H5C	109.5	H24A—C24—H24B	107.8
N1—C6—C7	112.8 (7)	C24—C25—C26	109.0 (8)
N1—C6—C11	110.2 (6)	C24—C25—H25A	109.9
C7—C6—C11	109.5 (6)	C26—C25—H25A	109.9
N1—C6—H6	108.1	C24—C25—H25B	109.9
C7—C6—H6	108.1	C26—C25—H25B	109.9
C11—C6—H6	108.1	H25A—C25—H25B	108.3
C8—C7—C6	110.6 (8)	C27—C26—C25	111.6 (7)
C8—C7—H7A	109.5	C27—C26—H26A	109.3
C6—C7—H7A	109.5	C25—C26—H26A	109.3
C8—C7—H7B	109.5	C27—C26—H26B	109.3
C6—C7—H7B	109.5	C25—C26—H26B	109.3
H7A—C7—H7B	108.1	H26A—C26—H26B	108.0
C7—C8—C9	112.6 (7)	N4—C27—C26	110.5 (7)
C7—C8—H8A	109.1	N4—C27—C22	110.6 (7)
C9—C8—H8A	109.1	C26—C27—C22	109.7 (8)
C7—C8—H8B	109.1	N4—C27—H27	108.7
C9—C8—H8B	109.1	C26—C27—H27	108.7
H8A—C8—H8B	107.8	C22—C27—H27	108.7
C8—C9—C10	110.3 (7)	C29—C28—H28A	109.5
C8—C9—H9A	109.6	C29—C28—H28B	109.5
C10—C9—H9A	109.6	H28A—C28—H28B	109.5
C8—C9—H9B	109.6	C29—C28—H28C	109.5
C10—C9—H9B	109.6	H28A—C28—H28C	109.5
H9A—C9—H9B	108.1	H28B—C28—H28C	109.5
C11—C10—C9	111.2 (8)	N4—C29—C30	118.8 (8)
C11—C10—H10A	109.4	N4—C29—C28	118.7 (7)
C9—C10—H10A	109.4	C30—C29—C28	122.5 (8)
C11—C10—H10B	109.4	C29—C30—C31	124.5 (9)
C9—C10—H10B	109.4	C29—C30—Br4	118.5 (7)
H10A—C10—H10B	108.0	C31—C30—Br4	116.9 (7)
N2—C11—C10	112.1 (7)	O4—C31—C30	121.2 (9)
N2—C11—C6	108.1 (6)	O4—C31—C32	119.2 (11)
C10—C11—C6	109.6 (6)	C30—C31—C32	119.5 (11)
N2—C11—H11	109.0	C4—N1—C6	127.2 (7)
C10—C11—H11	109.0	C4—N1—H1	116.4
C6—C11—H11	109.0	C6—N1—H1	116.4
C13—C12—H12A	109.5	C13—N2—C11	125.1 (7)
C13—C12—H12B	109.5	C13—N2—H2	117.5
H12A—C12—H12B	109.5	C11—N2—H2	117.5
C13—C12—H12C	109.5	C20—N3—C22	127.1 (7)
H12A—C12—H12C	109.5	C20—N3—H3	116.5
H12B—C12—H12C	109.5	C22—N3—H3	116.5
N2—C13—C14	117.6 (8)	C29—N4—C27	125.3 (7)
N2—C13—C12	117.4 (8)	C29—N4—H4	117.3
C14—C13—C12	124.9 (8)	C27—N4—H4	117.3
			
O1—C2—C3—C4	−9.1 (14)	N3—C22—C23—C24	179.2 (8)
C1—C2—C3—C4	168.9 (9)	C27—C22—C23—C24	−57.2 (10)
O1—C2—C3—Br1	179.5 (7)	C22—C23—C24—C25	56.6 (12)
C1—C2—C3—Br1	−2.6 (12)	C23—C24—C25—C26	−54.1 (12)
C2—C3—C4—N1	6.0 (13)	C24—C25—C26—C27	55.3 (12)
Br1—C3—C4—N1	177.2 (6)	C25—C26—C27—N4	−179.2 (8)
C2—C3—C4—C5	−176.5 (8)	C25—C26—C27—C22	−57.0 (11)
Br1—C3—C4—C5	−5.3 (11)	N3—C22—C27—N4	−54.4 (8)
N1—C6—C7—C8	178.6 (7)	C23—C22—C27—N4	180.0 (7)
C11—C6—C7—C8	−58.3 (10)	N3—C22—C27—C26	−176.5 (6)
C6—C7—C8—C9	56.6 (11)	C23—C22—C27—C26	57.9 (9)
C7—C8—C9—C10	−54.3 (11)	N4—C29—C30—C31	2.1 (12)
C8—C9—C10—C11	55.0 (10)	C28—C29—C30—C31	−178.6 (8)
C9—C10—C11—N2	−178.1 (7)	N4—C29—C30—Br4	178.1 (6)
C9—C10—C11—C6	−58.0 (9)	C28—C29—C30—Br4	−2.5 (11)
N1—C6—C11—N2	−53.7 (9)	C29—C30—C31—O4	−3.4 (15)
C7—C6—C11—N2	−178.3 (7)	Br4—C30—C31—O4	−179.5 (8)
N1—C6—C11—C10	−176.2 (7)	C29—C30—C31—C32	172.3 (9)
C7—C6—C11—C10	59.2 (10)	Br4—C30—C31—C32	−3.8 (12)
N2—C13—C14—C15	6.2 (13)	C3—C4—N1—C6	−161.2 (8)
C12—C13—C14—C15	−176.2 (8)	C5—C4—N1—C6	21.2 (11)
N2—C13—C14—Br2	178.1 (6)	C7—C6—N1—C4	−132.1 (8)
C12—C13—C14—Br2	−4.3 (11)	C11—C6—N1—C4	105.2 (8)
C13—C14—C15—O2	−10.3 (14)	C14—C13—N2—C11	−164.3 (7)
Br2—C14—C15—O2	177.7 (7)	C12—C13—N2—C11	17.9 (11)
C13—C14—C15—C16	168.6 (10)	C10—C11—N2—C13	−131.6 (8)
Br2—C14—C15—C16	−3.4 (12)	C6—C11—N2—C13	107.4 (8)
O3—C18—C19—C20	−10.1 (14)	C19—C20—N3—C22	−159.0 (8)
C17—C18—C19—C20	168.3 (9)	C21—C20—N3—C22	22.0 (12)
O3—C18—C19—Br3	176.3 (7)	C23—C22—N3—C20	−128.5 (8)
C17—C18—C19—Br3	−5.2 (12)	C27—C22—N3—C20	107.0 (8)
C18—C19—C20—N3	4.9 (13)	C30—C29—N4—C27	−159.8 (8)
Br3—C19—C20—N3	178.4 (6)	C28—C29—N4—C27	20.9 (12)
C18—C19—C20—C21	−176.1 (8)	C26—C27—N4—C29	−129.6 (8)
Br3—C19—C20—C21	−2.6 (11)	C22—C27—N4—C29	108.7 (9)

### Hydrogen-bond geometry (Å, °)

**Table:**

*D*—H···*A*	*D*—H	H···*A*	*D*···*A*	*D*—H···*A*
N1—H1···O1	0.86	1.96	2.588 (8)	129
N2—H2···O2	0.86	1.93	2.584 (9)	131
N3—H3···O3	0.86	1.98	2.602 (9)	129
N4—H4···O4	0.86	1.97	2.596 (9)	129
C5—H5C···O2^i^	0.96	2.66	3.463 (12)	142
C12—H12B···O1^ii^	0.96	2.56	3.416 (12)	149
C23—H23A···O3^iii^	0.97	2.66	3.581 (12)	159
C28—H28C···O4^iv^	0.96	2.65	3.419 (13)	138

Symmetry codes: (i) −*x*+1, *y*−1/2, −*z*+1; (ii) −*x*, *y*+1/2, −*z*+1; (iii) −*x*+1, *y*+1/2, −*z*; (iv) −*x*+2, *y*−1/2, −*z*.
